# Perioperative management of elderly patients with osteoarthritis requiring total knee arthroplasty

**DOI:** 10.1177/1750458920936940

**Published:** 2021-03-21

**Authors:** Jiang An Lim, Azeem Thahir

**Affiliations:** 1 Department of Trauma and Orthopeadics, Addenbrookes Major Trauma Unit, Cambridge University Hospitals, Cambridge, UK; 2 School of Clinical Medicine, University of Cambridge, Cambridge, UK

**Keywords:** Total knee arthroplasty / Perioperative management / Osteoarthritis / Elderly / Orthopaedic surgery

## Abstract

Knee osteoarthritis is a common arthritic disease which mainly affects the elderly (≥65 years old) population. As a result of the cartilage degeneration, it can cause a significant amount of pain and functional limitation. In patients who are refractory to conservative management, total knee arthroplasty is being utilised as the last resort in management. In this review, we discuss the perioperative management of elderly patients with osteoarthritis requiring total knee arthroplasty.

**Provenance and Peer review:** Unsolicited contribution; Peer reviewed; Accepted for publication 3 June 2020.

## Introduction

Osteoarthritis (OA) is a degenerative disease which affects the weight-bearing joints, preventing individuals from participating in activities of daily living and work (Palmer 2012). OA of the knee causes significant pain along with deterioration in function and mobility, with the prevalence being higher in athletes and the elderly (≥65 years old) (Madaleno et al 2018). With an ageing population, this has resulted in an increase in both its incidence and prevalence, which is a major public health problem (Madaleno et al 2018). It is a leading cause of physical disability in the elderly and brings both individual and societal consequences.

There is currently no treatment that can adequately halt or reverse the progress of OA, with the mainstay of treatment being conservative lifestyle advice, pharmacological analgesia and appropriately timed surgical interventions (McAlindon et al 2014). In patients that do not respond to non-operative measures, primary total knee arthroplasty (TKA) is indicated and used to treat advanced osteoarthritis in one or more of the three knee compartments. TKA is a safe and highly effective treatment for moderate to severe osteoarthritic symptoms, and has shown to improve pain as well as restore function (Talmo et al 2012).

Despite its success in improving the quality of life in patients, TKA is associated with considerable acute postoperative pain, which if unrelieved may result in prolonged hospital stay and poorer functional outcomes (Buvanendran et al 2010). Furthermore, elderly patients are usually at higher risk of postoperative medical complications, but these can be avoided by following the enhanced recovery after surgery (ERAS) protocol, which is a multimodal perioperative care pathway designed to achieve early recovery for patients undergoing major surgery (Ghosh & Chatterji 2019).

The final objective for patients after TKA is to lead an active life, free from pain. This outcome is dependent on the operation along with the steps taken before and after the surgery in the perioperative phase to minimise any complications as well as to speed up the recovery process (Sakellariou et al 2015). Therefore, this review will discuss the perioperative management of elderly patients with osteoarthritis undergoing TKA, which must be familiar to all healthcare providers involved in the care of these patients.

## Preoperative management

### Preoperative patient education

Patient education is a crucial component of preoperative management, which involves informing patients about the physical effects of TKA, changes to anatomical ability post-surgery as well as the benefits and risks of TKA (Ghosh & Chatterji 2019). Furthermore, it is also important to manage the patient’s expectation regarding the surgery, making sure that functional outcomes expected from TKA are realistic. They need to be aware of the timing of surgery in relation to the waiting list as this can vary from three to six months depending on the hospital.

### Preoperative assessment

Obesity is a prominent risk factor for developing osteoarthritis by exerting deleterious effects on joints through both biomechanical and systemic inflammatory changes (NICE 2014). It also puts patients at an increased risk of complications such as deep periprosthetic joint infections after surgery (Springer et al 2017). With evidence showing that obese patients have less improvement in outcomes after TKA and that delaying surgery by eight months does not worsen their final outcomes, it has been suggested that TKA should be delayed in obese patients to allow them to lose weight (Judge et al 2012, Tuominen et al 2010).

The systematic use of validated scales preoperatively such as the Oxford Knee Score, which is a patient-reported outcome measure specifically designed to assess knee function and pain before and after TKA, should be encouraged as it enables assessment of outcomes after TKA (BOA 2017).

### Preoperative analgesia

Preemptive analgesia is an analgesia strategy that uses analgesics before operation, which has been demonstrated to decrease postoperative analgesic consumption while exhibiting better analgesic effects (Lee et al 2015). Preoperative celecoxib as analgesia was found to be more efficient and equally tolerated compared to postoperative celecoxib in patients with knee osteoarthritis undergoing TKA (Liu & Wang 2018). Patients consumed less patient-controlled analgesia, had decreased pain Visual Analogue Scale (VAS) scores with similar number of adverse events and showed greater active flexional and passive flexional angle at 72 hours. Therefore, preoperative celecoxib not only exhibited better analgesia efficacy, but also improved knee function and promoted rehabilitation of patients.

The increased utilisation of opioids has been accompanied by concerns about their adverse effects on surgical outcome when taken prior to surgery. High doses of opioids have been associated with the development of opioid dependence and hyperalgesia, which could contribute to intractable pain (Goesling et al 2016). Patients taking opioids prior to TKA experienced less pain relief six months postoperatively than patients who had not used opioids prior to TKA, have higher rates of revision for residual pain or stiffness and worse functional outcomes within the first week (Aasvang et al 2016).

The administration of multiple doses of preoperative dexamethasone has been shown to improve clinical outcomes after TKA (Xu et al 2018). Multiple doses of dexamethasone were found to reduce postoperative pain, decrease consumption of analgesic drugs, provide more powered inflammation control and prevent postoperative nausea and vomiting with no difference in postoperative serum glucose levels in comparison to single-dose administrations (Xu et al 2018).

### Preoperative rehabilitation

Prehabilitation, which is the concept of preoperative physiotherapy and exercise programmes has been proposed as a potential way to expedite recovery times (Casana et al 2019). It has shown to be effective in reducing the length of hospital stay, improving knee range of motion and sit to stand test as well as improving postural control after TKA (Casana et al 2019). Since preoperative quadriceps strength is a strong predictor of functional performance two years after TKA and is inversely related to knee pain, it would be prudent to employ prehabilitation exercises in patients before TKA (Amin et al 2009).

Aside from mechanically based strength training, it has been shown that metabolic stimuli have the ability to counteract skeletal muscle atrophy (Kubota et al 2008). Therefore, blood flow restriction exercises have been proposed as a prehabilitation concept, which achieves muscular hypertrophy through low resistance training combined with a suppression of venous blood flow in an extremity (Franz et al 2018). Blood flow restriction may then allow patients who are unable to go through high-resistance exercises because of massive pain caused by advanced joint degeneration to switch from a mechanical to a metabolic stress, which favours muscle adaptations preoperatively. Blood flow restriction also did not negatively affect arterial stiffness in older adults, but instead improved vascular endothelial function and peripheral blood circulation in older people, which may be useful in patients with cardiovascular co-morbidities (Pinto et al 2018).

## Intraoperative management

### Patient safety and planning

Before carrying out any surgical procedure, it is imperative that the World Health Organization (WHO) checklist is performed in the anaesthetic room as well as the operating room where the identity of the patient is confirmed along with the surgical site marking as per documentation (WHO 2009). Planning and discussion of TKA among every member of the operative team are imperative to allow the procedure to proceed as smoothly as possible. This includes checking that the knee implant is specific to the patient before beginning the surgery and making sure that the implant is firmly in place by requesting postoperative X-rays to identify periprosthetic loosening.

### Tourniquets

There are both advantages and disadvantages to the use of a tourniquet in TKA: tourniquets allow surgeons to obtain a clear visualisation of the operative field, reduce intraoperative blood loss and provide a cleaner field for cement penetration and fixation. However, the use of tourniquets may cause extensive rhabdomyolysis due to local muscle damage, nerve damage, delayed recovery, acute pain and need for analgesia (Ejaz et al 2014).

Non-tourniquet TKA surgeries are beneficial in preventing adverse effects such as DVT/PE, support early recovery of patients by maintaining muscle strength as well as decreasing pain and opioid consumption: more so in females compared to males (Kheir et al 2018). Therefore, surgeons need to discuss and choose between the reported techniques of tourniquet application in the context of practicability and utility within the assessment of every individual patient before TKA.

### Blood management

Large blood loss after TKA remains a concern of surgeons, with total blood loss reaching 1.5 L on average, which then requires transfusions (Sizer et al 2015). Allogenic blood transfusions might increase the risk of longer hospital stays, infectious disease transmission, immunologic reactions, haemolytic and anaphylactic reactions and increase mortality (Klein 2010). Identifying influential factors of surgical blood loss is an important step towards establishing an effective blood management strategy and reducing the need for perioperative blood transfusion. Since males suffer more surgical blood loss than females, perioperative blood management should be enhanced in male patients (Guerin et al 2007).

The use of intravenous tranexamic acid has been found to reduce the need of blood transfusions, as it decreases postoperative blood loss and reduces swelling (Hu et al 2018). On the other hand, intra-articular drains have no longer been recommended due to the lack of difference in outcome measures (Maniar et al 2019). Furthermore, it has been shown that perioperative intravenous iron supplementation can reduce transfusion rates after TKA and in combination with intra-articulation administration of tranexamic acid, reduces the rate of allogenic transfusions in patients undergoing bilateral TKA (Suh et al 2017). Therefore, intravenous iron supplementation is recommended to reduce the transfusion rate in patients with predictable blood loss during TKA.

## Postoperative management

### Pain management

Managing a patient’s response to the stress of surgery can increase the chance of a favourable postoperative outcome. Acute postoperative pain may be a direct consequence of the operation or anaesthetic protocol; therefore, a multimodal drug cocktail injection which includes gabapentin, paracetamol and oxycodone as proposed by the ERAS protocol to the periarticular region can assist with analgesia in the initial postoperative period (Soffin & YaDeau 2016). Peripheral nerve blockade has also been shown to reduce both pain and opioid requirements postoperatively as well as speed up recovery times (Moghtadaei et al 2014).

Local anaesthetics are also a core component of the multimodal pain management pathway, which seeks to relieve postsurgical pain with fewer side effects to facilitate recovery (Parvizi & Bloomfield 2013). Conventional local anaesthetics are limited by their short duration of analgesia; however, local infiltration analgesia with liposomal bupivacaine allows for prolonged release of bupivacaine, which can extend the local duration of pain control (Hu et al 2013). This then reduces the requirements for opioids, which improves early outcomes after TKA (Dysart et al 2019).

Platelet-rich plasma injections that stimulate the natural healing cascade at the site of treatment were found to promote a favourable environment for joint tissue healing after TKA with a reduction in pain and improvement in knee function (Muchedzi & Roberts 2018). However, there is still a lack of standardisation in terms of platelet concentrations and frequency of injections for optimal efficacy as well as a limited number of randomised controlled trials performed.

### Postoperative rehabilitation

Accelerated physiotherapy regimens, where the patient is mobilised within 24 hours of surgery are the most beneficial active physiotherapy interventions during the acute hospital stay following TKA (Henderson et al 2018). It reduces both the length of stay in hospital and pain scores and improves function compared to starting physiotherapy a day later. Continuous passive motion for early joint mobilisation after TKA has been shown to be beneficial due to preventing haemarthrosis, peri-articular oedema and joint stiffness, which improves the range of motion (Sanchez Mayo et al 2015).

It is well known that muscle strength and function are reduced for a long period postoperatively with a reduction of up to 80% in knee extension strength after TKA (Holm et al 2010). Quadricep muscle strength is crucial for both functional performance and the survival of the artificial implant, with the loss of muscle strength associated with an increased risk of falling in the elderly (Roos et al 2011). Therefore, effective strength training methods, such as maximal strength training, which have shown superior increases in knee extension muscle strength compared to those managed with standard rehabilitation should be applied to counter the reduced muscle strength (Skoffer et al 2016).

Postoperative limb positioning has been found to be a simple way to reduce blood loss and improve range of motion following TKA. Compared to other methods to reduce bleeding, which requires the use of dedicated instrumentations and risk of adverse drug reactions, a consistent limb position regime for 48–72 hours postoperatively represents an inexpensive and simple approach. Placing the knee in a flexion position has been shown to be effective in reducing total blood loss compared to when in extension; however, there is still no consensus regarding the optimal flexion position. Mild-flexion positions were superior in decreasing hidden blood loss, while high-flexion positions were superior in reducing transfusion requirements and improving postoperative range of motion (De Fine et al 2017, Fu et al 2016). However, placing the knee in the high-flexion position causes more patient discomfort, wound complications and increases the risk of residual fixed flexion deformity (Wu et al 2017).

### Complications

Early anti-coagulation is essential for the prevention of thrombosis, but the benefits may be offset by an increased risk of bleeding because of the hyperfibrinolysis (Gomez-Outes et al 2012). In hospitals, the risk of developing venous thromboembolism is higher than bleeding, suggesting a favourable risk profile for venous thromboembolism prophylaxis (Vekeman et al 2012). Therefore, the management of anti-coagulation and postoperative bleeding requires a delicate balance.

Other complications include postoperative delirium, nausea, vomiting, periprosthetic loosening and joint infections. A special emphasis should be placed on the early detection of infection as it can compromise the viability of the implant as well as cause chronic pain (Mandalia et al 2008). Therefore, a multidisciplinary team discussion immediately after TKA between all health care professionals involved in the patient’s care is essential for early detection of any complications and to ensure early and safe discharge.

## Conclusion

To conclude, knee osteoarthritis causes significant pain which requires surgical intervention when non-responsive to conservative management. Optimal perioperative management of these patients has the potential to improve recovery and prevent the development of chronic pain, which is crucial to ensure a successful outcome and to limit complications.

## Key Phrases

1. Knee osteoarthritis causes significant pain and is commonly found in the elderly population.
2. Total knee arthroplasty is indicated in patients who do not respond to conservative management.
3. Prehabilitation found to expedite recovery time.
4. Intravenous tranexamic acid administration reduces the need for blood transfusion.
5. Accelerated physiotherapy regimens are effective in improving outcomes.
